# Knowledge of Gestational Diabetes Mellitus Among Saudi Women in a Primary Health Care Center of Almadinah Almunawarah, Kingdom of Saudi Arabia

**DOI:** 10.7759/cureus.22979

**Published:** 2022-03-08

**Authors:** Abeer Abdulaziz Khayat, Nahid fallatah

**Affiliations:** 1 Family Medicine, Academy of Family Medicine/Ministry of Health, Al-Madinah, SAU

**Keywords:** kingdom of saudi arabia (ksa), pregnant women, oral glucose tolerance test, awareness, gestational diabetes

## Abstract

Background

Gestational diabetes mellitus (GDM) is a type of diabetes mellitus known as any stage of glucose intolerance with onset or first recognition during pregnancy. Awareness of GDM is the first step toward its screening in pregnancy. This study was designed to assess knowledge of GDM, its screening, and risk factors among Saudi women attending primary healthcare center in Almadinah Almunawarah, Kingdom of Saudi Arabia.

Methods

This was an observational cross-sectional study conducted on Saudi women who attended the primary healthcare centers in Almadinah Almunawarah during the study period from January 2021 to June 2021. The sampling technique used was the stratification of primary healthcare centers in Madinah. According to the Epi-Info, version 3.5.1, the minimum sample size was 292. Data collection was done using a valid, Arabic self-administered questionnaire, which was composed of two main parts: general sociodemographic data and a questionnaire to assess GDM knowledge and awareness (12 questions). Data was recorded and analyzed using SPSS version 26.

Results

In this study, 333 women were enrolled with an age range between 18 and 60 years, with a mean of 34.31±9.21 years. Overall, more than half of the women (53.45%) had a poor level of knowledge related to GDM, whereas only 7.80% had a good level of knowledge. Results of multivariate logistic regression analysis revealed that women living in rural areas were at almost four-fold higher risk of having a poor level of knowledge (adjusted odds ratio (aOR): 3.97; 95% confidence interval (CI): 1.44-41.98, p=0.0031). With a one-year increase in women’s age, the risk of poor knowledge increased by 4% (aOR: 1.38; 95% CI: 1.08-1.48, p=0.001). In comparison to illiterate women, university-graduated and postgraduate women had a significantly lower risk of poor knowledge (aOR: 0.03; 95% CI: 0.01-0.31, p=0.001 and aOR: 0.19; 95% CI: 0.06-0.66, p = 0.011, respectively).

Conclusion

The GDM knowledge of Saudi adult women was poor, particularly regarding risk factors, diagnosis, and treatment with insulin. However, their knowledge regarding treatment by lifestyle and diet modifications was quite acceptable.

## Introduction

Diabetes mellitus (DM) is widely recognized as one of the most deadly diseases endangering global public health. Every five minutes, two people die of diabetes-related causes and 14 adults are newly diagnosed, according to the Centers for Disease Control and Prevention (CDC) [1]. The International Diabetes Federation reported 3.4 million cases of DM in Saudi Arabia in 2015. The disease’s predicted cost in the country was $900 million in 2010 and is likely to rise to more than $6.5 billion by 2022 [2]. GDM is a kind of diabetes mellitus defined as any stage of glucose intolerance that develops during pregnancy or is first recognized during pregnancy, it complicates over 7% of all pregnancies, resulting in more than 200,000 cases per year [3].

The prevalence of GDM may range from 1 to 14% of all pregnancies, depending on population studies and diagnostic tests used [3]. GDM usually has no symptoms, although it can cause excessive urination, nausea, vomiting, lethargy, bladder infections, and yeast infections [4]. Uncontrolled glycemia during pregnancy may have negative consequences for both the mother and the fetus [5]. Maternal side effects include cesarean section, abortion, polyhydramnios, pre-eclampsia, premature labor, placenta previa, urinary tract infection, pruritus vulvae, puerperal sepsis, and pyelonephritis [5,6]. Maternal GDM is linked to both macrosomia and the pathophysiological effects of fetal hyperglycemia and hyperinsulinism, posing a risk to the fetus and newborn [7]. When compared to the general population, mothers with GDM have a higher risk of developing type 2 diabetes mellitus (T2DM) [4]. Preventing the progression of GDM may reduce the prevalence of T2DM and long-term morbidity in babies born to mothers with the disease [4].

Furthermore, focusing on women’s health and achieving a better understanding of their health is one of the primary aims of the World Health Organization (WHO) and the Saudi Kingdom’s 2030 vision. Research on the awareness of GDM among pregnant women has been undertaken in various parts of Saudi Arabia, but no previous study has been conducted in the Almadinah Almunawarah region. The goal of this study is to assess Saudi women’s understanding of GDM in a primary health care center (PHCC) in Almadinah Almunawarah, Saudi Arabia.

## Materials and methods

This was an observational cross-sectional study conducted in Almadinah Almunawarah in the Academy of Family Medicine, Ministry of Health (MOH) from January 2021 to June 2021. Almadinah is located in the western Saudi Arabian province of Hejaz. Almadinah Almunawarah has five hospitals, each of which is signed to have a group of PHCCs, for a total of 54 PHCCs. All women over the age of 18, married or unmarried, who attended PHCCs were included. The sample size was calculated according to the Epi-Info, version 3.5.1 by taking the expected frequency of awareness as 50%, the worst acceptable frequency as 46%, and the confidence interval (CI) of 95%. After adding 15% of the non-response rate the minimum sample size was 292, however, 333 participants were included during the study period.

Based on the most recent MOH update in Almadinah Almunawarah, data was obtained using a multistage technique, with stage 1 stratifying PHCCs according to which hospital is signed in and stage 2 involved selecting two PHCCs at random from each hospital group using the Quraa app. In the third step, 35 people were chosen at random from each PHCC.

Data was collected after the proposal was approved by Institutional Review Board, General Directorate of Health Affairs, MOH, Almadinah (approval number: H-03-M-084). The researcher distributed the questionnaire herself and recruited students to serve as data collectors. They were well-trained to answer any follow-up questions from participants after they had given their consent. To increase the response rate, the researcher gathered the questionnaires at the same time. Confidentiality of data was maintained throughout the project.

The questionnaires have 12 questions with yes, no, and don’t know answers. Each accurate response was given a score of one, and each woman was given a score out of a possible 12. “Poor knowledge” was defined for a score of 0-4, “fair knowledge” for a score of 5-8, and “excellent GDM knowledge” for a score of 9-12. Participants were then separated into two groups: those with weak knowledge and those with fair/good knowledge.

The researcher entered the questionnaire data into Microsoft Excel and analyzed it with the Statistical Package for the Social Sciences (SPSS) version 26 (IBM Corp., Armonk, USA). Categorical variables were described using frequencies and percentages, whereas numerical variables were described using mean and standard deviation (SD). The chi-square test was used to study the association between categorical variables and Fischer’s exact test was used instead of the chi-square test in the case of small frequencies. The student’s t-test was applied to compare the means of a continuous variable between two different groups. Multivariate logistic regression analysis was applied to control for the confounding effect and the results were expressed as an adjusted odds ratio (aOR) and a 95% CI. A p-value ≤ 0.05 was considered statistically significant.

## Results

The study included 333 women, with ages ranging between 18 and 60 years. The mean age was 34.31±9.21 years. Demographic data is summarized in Table 1. The majority of participants 321 (96.3%) live in urban areas, and more than half of them (178 (53.4%)) had more than one pregnancy/delivery. The majority 176 (52.85%) were university graduates and 211 (63.3%) were married. More than one-quarter of them (28.22%) work in the medical field, whereas 103 (30.3%) work in non-medical fields.

**Table 1:** Demographic characteristics of the participants (n=333)

Variables	Frequency	Percentage (%)
Residence		
Urban	321	96.3
Rural	12	3.6
Number of pregnancies/deliveries		
None	92	27.6
Once	63	18.9
More than once	178	53.4
Educational level		
Illiterate	25	7.5
Secondary school	102	30.6
University	176	52.85
Postgraduate	30	9.90
Marital status		
Single	66	19.8
Married	211	63.3
Divorced	50	15.0
Widowed	6	1.80
Job type		
Not working	136	40.8
Medical field	94	28.22
Non-medical field	103	30.9
Somebody in the family working in the medical field		
No	210	63.0
Yes	123	36.9

Regarding their BMI, overweight and obese were observed among 99 (29.72%) and 87 (26.12%) of the participants, respectively. However, 121 (36.33%) had normal BMI, while only 26 (7.80%) were underweight. A history of diabetes was reported by 155 (46.54%) of the participants. A previous history of GDM was mentioned by 54 (16.21%) of the women. A history of chronic diseases was reported by more than one-third of the participants 130 (39.03%). 50.21% of people have dyslipidemia, followed by hypertension (46.39%) and type 2 diabetes (32.41%).

Table 2 summarizes the responses of the participants to the GDM knowledge statements. More than half of them (196 (58.85%)) could identify that lifestyle and dietary changes are part of the GDM management plan, that GDM increases the risk of future T2DM 188 (56.45%), that untreated GDM increases the risk of neonatal complications 174 (52.25%), and that a prior personal history of GDM increases the risk of future GDM, 168 (50.6%). On the other hand, less than a quarter (81 (24.32%)) of them properly knew that increasing the number of pregnancies increases the risk of developing GDM. Insulin is one of the appropriate GDM management plans. In the absence of risk factors, the oral glucose tolerance test (OGTT) is the gold standard test to screen for GDM (71 (21.32%)) and only 52 (15.61%) knew the optimal time to do OGTT, which is between 24 and 28 weeks.

**Table 2:** Response of the women to knowledge statements about gestational diabetes (n=333) GDM: gestational diabetes mellitus; OGTT: oral glucose tolerance test

	Correct answers	
	No	%
Increase the number of pregnancies increases the risk of developing GDM	81	24.32
Weight gain preconception increases the risk of developing GDM	133	39.93
Excessive weight gain in pregnancy increase the risk of future GDM	121	36.33
Prior personal history of GDM increases the risk of future GDM	168	50.45
The family history of GDM increases the risk of future GDM	105	31.53
What is the optimal time to do OGTT, in absence of risk factors (24-28 weeks)	52	15.61
Lifestyle and diet modifications are part of the GDM management plan	196	58.85
Insulin is one of the appropriate GDM management planes	75	22.52
GDM usually disappears after delivery	164	49.24
Untreated GDM increases the risk of neonatal complications	174	52.25
GDM increases the risk of future type 2 diabetes	188	56.45
OGTT is the gold stander test to screen for GDM	71	21.32

Overall, more than half of the women (178 (53.45%)) had a poor level of knowledge related to GDM, whereas only 26 (7.80%) had a good level of knowledge. However, 129 (38.73%) had fair knowledge. Women living in urban areas were more likely to have fair/good GDM knowledge compared to those living in rural areas (48.59% versus 25%), p=0.016. The age of women with fair/good knowledge was significantly lower than that of those with poor knowledge (33.05±7.19 versus 39.21±10.21, p<0.001. About two-thirds (68.75%) of university-graduated women compared to only 8% of illiterate and 20.58% of secondary school or less educated women expressed fair/good levels of GDM knowledge, p<0.001. More than half of single (53.03%) and married (51.65%) women, compared to 16.66% of widowed women, had fair/good GDM knowledge, p<0.001. Women who have somebody in their family working in a medical field were more likely to express a higher level of fair/good GDM knowledge than their peers (57.72% versus 40.95%), p=0.001 (Table 3).

**Table 3:** Association between demographic characteristics of the participants and their level of gestational diabetes knowledge *Chi-square test ⱶFischer’s Exact test **Student’ t- test

	GDM knowledge level		p-value*
	Poor n=178	Fair/Good n=155	
Residence			0.0161ⱶ
Urban (n=321)	165 (51.40%)	156 (48.59%)	
Rural (n=12)	9(75%)	3 (25%)	
Age (years) Mean±SD	39.21±10.21	33.05±7.19	<0.001
Number of pregnancies/deliveries			0.879*
None (n=92)	47 (51.08%)	45 (48.91%)	
Once (n=63)	33 (52.38%)	30 (47.61%)	
More than once (n=178)	96 (53.93%)	82 (46.06 %)	
Educational level			<0.001*
Illiterate (n=25)	23 (92%)	2 (8%)	
secondary school (n=102)	81 (79.41%)	21 (20.58%)	
University (n=176)	55 (31.25%)	121 (68.75%)	
Postgraduate (n=30)	15 (50.0%)	15 (50.0%)	
Marital status			<0.001*
Single (n=66)	31 (46.96%)	35 (53.03%)	
Married (n=211)	102 (48.34%)	109 (51.65%)	
Divorced (n=50)	38 (76%)	12 (24%)	
Widowed (n=6)	5 (83.33%)	1 (16.66%)	
Job place			0.249*
Not working (n=136)	75 (55.14%)	61 (44.85%)	
Medical field (n=94)	44 (46.80%)	50 (53.19%)	
Non-medical field (n=103)	57 (55.33%)	46 (44.66%)	
Somebody in the family working in the medical field			0.001*
No (n=210)	124 (59.04%)	86 (40.95%)	
Yes (n=123)	52 (42.27%)	71 (57.72%)	

Results of multivariate logistic regression analysis revealed that women living in rural areas were at almost four-fold higher risk of having a poor level of knowledge aOR: 3.97; 95% CI:1.44-41.98, p=0.0031). With a one-year increase in women’s age, the risk of poor knowledge increased by 4% (aOR: 1.38; 95% CI: 1.08-1.48, p=0.001). Considering underweight women as a reference category, obese women were at 80% lower risk of having poor knowledge (aOR: 0.34; 95% CI: 0.04-0.52, p=0.000). Women who had a history of knowing someone who had gestational diabetes had a significantly lower risk of having poor knowledge (aOR: 0.49; 95% CI: 0.22-0.79, p=0.0289). Similarly, women with a family member working in a medical field had a significantly lower risk of poor knowledge (aOR: 0.47; 95% CI: 0.30-0.89, p=0.008). University-graduated and postgraduate women had a significantly lower risk of having poor knowledge compared to illiterate women (aOR: 0.03; 95% CI: 0.01-0.31, p=0.001 and aOR: 0.19; 95% CI: 0.06-0.66, p=0.011, respectively). Marital status, history of chronic diseases, and family history of diabetes were not significantly associated with a poor level of GDM knowledge (Table ).

**Table 4:** Predictors of poor gestational diabetes mellitus knowledge among the participants: multivariate logistic regression analysis

Variables	Adjusted odds ratio	95% confidence interval	p-value
Residence			0.031
Urban (n=321)	1.0	-	
Rural (n=12)	3.97	1.44-41.98	
Age (years)	1.38	1.08-1.48	0.001
Body mass index			
Underweight (n=26)	1.0	-	-
Normal (n=121)	0.84	0.26-2.61	0.854
Overweight (n=99)	0.41	0.14-1.22	0.246
Obese (n=87)	0.34	0.04-0.52	0.000
History of ever knowing anybody affected by gestational diabetes			
No (n=151)	1.0	-	0.0289
Yes (n=182)	0.49	0.22-0.79	
Somebody in the family working in the medical field			
No (n=210)	1.0	-	0.008
Yes (n=123)	0.47	0.30-0.89	
Educational level			
Illiterate (n=25)	1.0	-	-
secondary school (n=102)	0.51	0.06-1.87	0.226
University (n=176)	0.03	0.01-0.31	<0.001
Postgraduate (n=30)	0.19	0.06-0.66	0.011

## Discussion

Limited studies have been undertaken to address the knowledge about gestational diabetes in Saudi Arabia, although it is not an uncommon health problem. Additionally, the first step toward screening for women with diabetes during pregnancy is assessing the awareness of women of reproductive age about early detection of GDM and its adverse outcome. Therefore, the present study was conducted to assess the knowledge of Saudi women attending PHCC in Almadinah Almunawarah, Kingdom of Saudi Arabia, about GDM, its screening, and associated factors.

Overall, GDM knowledge was poor among more than half of the women (53.45%) and good only among a minority of them (7.80%). Therefore, it needs improvement in most elements, particularly those related to risk factors, diagnosis, and the role of insulin in management. In Dhahran, 37% of antenatal women were aware that GDM could result in cases of low neonate birth weight, 62.7% were aware that GDM could result in DM, 24.9% were aware that GDM could result in an increased risk of congenital anomalies, and 50% were aware that the children of GDM mothers could develop DM in the future [8]. Alharthi et al. (2018) using the same questionnaire reported that most Saudi women had a fair level of knowledge; half of them were aware of the GDM risk factors (54%), while only 15.9% were aware of the GDM diagnosis [9]. Comparison between the current study and those studies should be done with caution due to the different demographic characteristics of the participants, although the results were quite similar. In Najran, Saudi Arabia (2019), most of the women thought that GDM had no impact on them (71.3%) and their neonates (65.2%).

In a similar recent study carried out in Nigeria (2020), the majority of the women of reproductive age were aware of DM, but only 38.2% knew that DM can happen during pregnancy. Good knowledge score rates ranged between 28.8% and 35.8% regarding GDM definition, risk factors, diagnosis, management, and complications. Overall, a good level of knowledge was observed among 26.2% of women [10]. In India (2018), an adequate level of knowledge about GDM and its risk factors was observed among 35.2% and 21.5% of the mothers who attended antenatal clinics, respectively [11]. In Bangladesh, the majority of the women (83.3%) were familiar with GDM; however, 60.7% had inadequate knowledge [12]. In Tunisia, 47% of pregnant women had no idea about GDM; however, 43% knew that GDM occurs in pregnancy [13]. The difference in the level of knowledge between various studies, including the present one, could be explained by using different tools to assess knowledge, in addition to variation in the demographic characteristics of the participants.

In the current study, after controlling for confounders in multivariate analysis, younger women, those living in urban areas, more educated women, women with obesity, those knowing anybody affected by GDM, and those having somebody in their family working in the medical field were more knowledgeable about GDM than their counterparts. Alharthi et al. (2018) reported that multigravida and women with a previous history of GDM were most aware of GDM. Participants who live in urban areas in the central region of Saudi Arabia, are married, work in the medical field, are more educated, and have personal and/or family histories of chronic diseases expressed a higher level of knowledge [14]. In Najran (Saudi Arabia), predictors of knowledge of the impacts of GDM were educational level, nationality, number of pregnancies, personal history of GDM, and chronic hypertension [9]. In a study carried out recently in Nigeria among women of reproductive age, residence in urban areas, being married, and having been pregnant were significant indicators for good GDM knowledge level [10]. In Norway (2019), non-native speakers were more likely to have poor knowledge of GDM compared to native speakers, and they attributed this to poor language skills [15]. In India, significant predictors for knowledge level were educational level and place of residence [11]. In another Indian study, more educated women and those living in urban areas were more knowledgeable about GDM [16].

Although the present study explored an important issue in our community, some limitations should be addressed. Among the important limitations is the cross-sectional design adopted in the study, as it proves only association and not causality between exposure and outcome variables. The second important limitation is the conduction of the study among women attending PHCCs only, which limits the study to those women, which could impact the generalizability of results. Finally, the collection of data from different PHCCs was difficult as a result of the long distance between them and limited time. However, recruiting and training some students to help with data collection may help us overcome this difficulty.

## Conclusions

Overall, the GDM knowledge of Saudi adult women was poor in more than half of them, particularly regarding risk factors, diagnosis, and treatment by insulin. However, their knowledge regarding treatment by lifestyle and diet modifications was quite acceptable. Younger women, those living in urban areas, more educated, obese, those knowing anybody affected by GDM, and those having somebody in their family working in the medical field were more knowledgeable about GDM than their counterparts.
